# Antioxidant Potential and Inhibition of Mitochondrial Permeability Transition Pore by Myricetin Reduces Aluminium Phosphide-Induced Cytotoxicity and Mitochondrial Impairments

**DOI:** 10.3389/fphar.2021.719081

**Published:** 2021-11-09

**Authors:** Ahmad Salimi, Zhaleh Jamali, Mohammad Shabani

**Affiliations:** ^1^ Department of Pharmacology and Toxicology, School of Pharmacy, Ardabil University of Medical Sciences, Ardabil, Iran; ^2^ Traditional Medicine and Hydrotherapy Research Center, Ardabil University of Medical Sciences, Ardabil, Iran; ^3^ Student Research Committee, School of Medicine, Shahroud University of Medical Sciences, Shahroud, Iran; ^4^ Students Research Committee, School of Pharmacy, Ardabil University of Medical Sciences, Ardabil, Iran

**Keywords:** cardiomyopathy, poisoning, flavonoids, antioxidant, mitochondrial dysfunction

## Abstract

Oxidative stress and mitochondrial dysfunction are involved in the mechanisms of cardiac toxicity induced by aluminum phosphide (AlP). AlP-induced cardiotoxicity leads to cardiomyocyte death, cardiomyopathy, cardiac dysfunction, and eventually severe heart failure and death. Importantly, protecting cardiomyocytes from death resulting from AlP is vital for improving survival. It has been reported that flavonoids such as myricetin (Myr) act as modifiers of mitochondrial function and prevent mitochondrial damage resulting from many insults and subsequent cell dysfunction. In this study, the ameliorative effect of Myr, as an important antioxidant and mitochondrial protective agent, was investigated in cardiomyocytes and mitochondria isolated from rat heart against AlP-induced toxicity, oxidative stress, and mitochondrial dysfunction. Treatment of AlP (20 μg/ml) significantly increased cytotoxicity; reduced glutathione (GSH) depletion, cellular reactive oxygen species (ROS) formation, malondialdehyde (MDA) level, ATP depletion, caspase-3 activation, mitochondrial membrane potential (ΔΨm) collapse, and lysosomal dysfunction; and decreased the activities of superoxide dismutase (SOD), catalase (CAT), and glutathione peroxidase (GSH-Px) in intact cardiomyocytes. Also, treatment of AlP (20 μg/ml) significantly increased mitochondrial dysfunction and swelling in isolated mitochondria. Myr (80 µM) appeared to ameliorate AlP-induced cytotoxicity in isolated cardiomyocytes; significantly lessened the AlP-stimulated intracellular ROS and MDA production and depletion of GSH; and increased the activities of SOD, CAT, and GSH-Px. Furthermore, Myr (40 and 80 µM) lowered AlP-induced lysosomal/mitochondrial dysfunction, ATP depletion, and caspase-3 activation. In the light of these findings, we concluded that Myr through antioxidant potential and inhibition of mitochondrial permeability transition (MPT) pore exerted an ameliorative role in AlP-induced toxicity in isolated cardiomyocytes and mitochondria, and it would be valuable to examine its *in vivo* effects.

## Introduction

Pesticide poisoning is a global public health problem, and one-third of the suicides in the world is due to self-poisoning (Gunnell et al., 2007). Pesticide poisoning causes more deaths than infections in some parts of developing countries (Eddleston et al., 2002). Due to pesticide poisoning, every year, more than 300,000 deaths occur in the world (Gurjar et al., 2011). The number of annual suicides worldwide due to pesticide self-poisoning is 110,000–168,000 cases (Dandona and Gunnell, 2021). Aluminum phosphide (AlP), organochlorine, and organophosphate compounds are commonly used pesticides around the world. As a common indoor and outdoor pesticide, AlP is used in developing countries, because it is effective, cheap, free from toxic residue, and without effect on seed viability (Gurjar et al., 2011). Due to its low-cost availability, AlP is extensively used as suicidal poison. In developing countries such as Iran and India, AlP is emerging as a common self-poisoning agent (Etemadi-Aleagha et al., 2015; Mehrpour et al., 2018). The toxic effects of AlP on different tissues are associated with phosphine (PH_3_) gas and oxidative stress (Mehrpour et al., 2012). Phosphine gas induces oxidative stress through mitochondrial dysfunction, inhibition of cytochrome *c* oxidase in mitochondria and enzymatic antioxidants such as reduced glutathione (GSH), superoxide dismutase (SOD), catalase (CAT), and glutathione peroxidase (GSH-Px) (Bumbrah et al., 2012). Mitochondrial dysfunction, inhibition of cellular respiration, and antioxidant enzymes such as CAT, GR, and SOD can produce free radicals, lipid peroxidation, and oxidative stress. These alterations will lead to cellular injury and cytotoxicity via oxidative stress in different tissues. Among the human tissues, cardiac tissue is more vulnerable to AlP-induced toxicity, oxidative stress, and mitochondrial dysfunction, because the heart is rich in mitochondria and low antioxidant capacity (Sciuto et al., 2016; Tapio, 2016). It has been reported that near 70% of deaths caused by AlP were attributable to cardiovascular disorders (Ames, 2010). The exact mechanism of AlP cardiotoxicity has not yet been determined, but previous studies suggest that mitochondrial dysfunction and oxidative stress play a major role. Therefore, mitochondrial protective agents and antioxidants may play an effective role in reducing cardiac toxicity induced by AlP.

Myricetin (Myr) is a natural flavonoid compound extracted from the leaves and bark of *Myrica rubra* (Jones et al., 2011). Also, Myr is found in many beverages and foods, including red wine (grapes), teas, vegetables, fruits, berries, and honey (Jones et al., 2011). The health benefits of Myr such as anti-inflammatory, antioxidant, antitumor, antimicrobial, cardioprotective, and other pharmacological effects have been thoroughly investigated over the last decade (Semwal et al., 2016). Recently, due to potential clinical impact of Myr on cardiovascular functions, its cardioprotective effect has attracted attention from the research community (Tran and Wang, 2019). Previous studies have been reported several cardioprotective effects of Myr on isoproterenol (ISO)-induced myocardial infarction, ischemia/reperfusion (I/R)-induced myocardial injury, and endotoxin-induced inflammatory myocardial injury (Wang et al., 2019). These studies suggest that Myr may display beneficial effects again cardiotoxicity induced by drugs and chemicals. Moreover, Myr is well known for its effective reduction of oxidative stress by providing antioxidant benefits (Park et al., 2016). It has been reported that Myr inactivates free radicals such as superoxide anion radical via single electron transfer to form an aryloxy radical (Chobot and Hadacek, 2011). The antioxidant effect of Myr was reported in various animal models and cell-based assays (Barzegar, 2016). In addition, it has been reported that Myr can protect cells from various insults that lead to mitochondria-mediated cytotoxicity, and previous studies demonstrated that this compound attenuates the progression of diseases and toxicity associated with mitochondrial dysfunction and oxidative stress (Lagoa et al., 2011). Due to above beneficial effects of Myr in the reduction of oxidative stress and mitochondrial dysfunction, in this study, we searched the effects of Myr against AlP-induced toxicity, oxidative stress, and mitochondrial dysfunction in isolated cardiomyocytes and mitochondria obtained from rat heart.

## Materials and Methods

### Animals

Cardiomyocytes were isolated form male Wistar rats (body weight 200–220 g and 8–9 weeks old), which were purchased from the Baqiyatallah University of Medical Sciences (Tehran, Iran) and allowed *ad libitum* access to tap standard rodent diet and water. The experimental animals received human care in compliance with the Guide for the Care and Use of Laboratory Animals approved by the Ethics Committee of the Ardabil University of Medical Sciences (Ardabil, Iran) with ethics code IR.ARUMS.REC.1397.236. The animals were anesthetized by intraperitoneal injection of combination of ketamine (50 mg/kg) and xylazine (10 mg/kg) and sacrificed by stunning and cervical dislocation. In this study, the male rats were selected due to AlP toxicity ratio in men to women of 2:1 (Moghadamnia, 2012).

### Chemicals

Fetal bovine serum (FBS), penicillin and streptomycin solution, Medium 199, Collagenase Type II (product number: C2-BIOC, Sigma), 2′,7′-dichlorofluorescin diacetate (product number: D6883, Sigma), creatine, Hanks’ Balanced Salt Solution (HBSS), potassium chloride, *N*-(2-hydroxyethyl)piperazine-*N*′-(2-ethanesulfonic acid) (HEPES), carnitine, rhodamine 123 (product number: R8004, Sigma), taurine (product number: T0625, Sigma), dimethyl sulfoxide (DMSO), Trypan Blue, bovine serum albumin (BSA), sucrose, d-mannitol, 2-amino-2-hydroxymethyl-propane-1,3-diol (TRIS), 2 monopotassium phosphate, ethylenediaminetetraacetic acid (EDTA), 3-(4,5-dimethylthiazol-2-yl)-2,5-diphenyltetrazolium bromide (MTT), sodium succinate, 3-morpholinopropane-1-sulfonic acid (MOPS), magnesium chloride, rotenone, acridine orange (AO), butylated hydroxytoluene (BHT), ethylene glycol-bis(β-aminoethyl ether) (EGTA), Coomassie Brilliant Blue, 5,5′-dithiobis(2-nitrobenzoic acid) (DTNB), Myr (product number: M6760, Sigma), and butylated hydroxytoluene (product number: PHR1117, Sigma) were purchased from Sigma (St. Louis, MO, USA). AlP with a purity of about 99% was gifted from the Samiran Company (Tehran, Iran). AlP was freshly prepared before use and dissolved in DMSO (0.05%).

### Solutions and Drugs

Creatine–carnitine–taurine medium (CCT medium) contained the following: 3.6 g of HEPES (25 mM), 655.5 mg of creatine (5 mM), 395.4 mg of carnitine (2 mM), 625.5 mg of taurine (5 mM), and 10 µM of cytosine β-d-arabinofuranoside, and pH was adjusted to 7.4 with NaOH (2 mM) in a sterile medium. Powell medium contained thee following: 6.43 g of NaCl (110 mM), 0.19 g of KCl (2.5 mM), 0.16 g of KH_2_PO_4_ (1.2 mM), 0.3 g of MgSO_4_ 7H_2_O (1.2 mM), 5.96 g of HEPES (25 mM), and 1.98 g of d-(+)-glucose monohydrate (10 mM) in Aqua sterile (double distilled water), and pH was adjusted to 7.4 with NaOH (2 mM) in a sterile medium. Calcium chloride (CaCl_2_) contained 100 mM of CaCl_2_. Mitochondrial isolation buffer contained the following: 225 mM of d-mannitol, 75 mM of sucrose, and 0.2 mM of EDTA, and pH was adjusted to 7.4 with NaOH (2 mM). Mitochondrial assay buffer contained the following: 10 mmol/L of NaCl, 140 mmol/L of KCl, 0.5 mmol/L of KH_2_PO_4_, 2 mmol/L of MgCl_2_, 0.5 mmol/L of EGTA, and 20 mmol/L of HEPES and supplemented with 10 mmol/L of succinate and 1 mg/ml of rotenone, and pH adjusted to 7.4. Mitochondrial swelling buffer contained the following: 140 mmol/L of KCl, 2 mmol/L of MgCl_2_, 10 mmol/L of NaCl, 0.5 mmol/L of KH_2_PO_4_, 20 mmol/L of HEPES, and 0.5 mmol/L of EGTA and supplemented with 1 mg/ml of rotenone, and pH was adjusted to 7.4. All AlP and Myr-containing solutions were prepared fresh before the experiments.

### Cardiomyocyte Isolation

Cardiomyocytes were isolated from the rat heart as previously described by Nippert et al. (2017)and Ahangari et al. (2020). Briefly, after deep anesthesia was administered to the animals, their hearts were explanted, washed with Powell medium, and cannulated via the aorta in the Langendorff perfusion system. Hearts were perfused with Powell medium at a constant flow rate of 10 ml/min with a peristaltic pump for approximately 5 min (37°C) to wash away the blood and then with 25 ml of warm Powell medium supplemented with collagenase (25 mg in 5 ml), and finally, the cardiac tissues were enzymatically dissociated during 25 min. Ventricles were then separated from the atria, cut in small pieces, and shaken for 10 min in 15 ml of warm Powell medium supplemented with collagenase in the presence of 50 μM of CaCl_2_. The isolated cardiomyocytes were suspended in CCT medium supplemented with 100 μg/ml of penicillin, 100 μg/ml of streptomycin, and 10% FBS in a humidified air containing 5% CO_2_ at 37°C.

### Experimental Design

The experimental groups were categorized into six groups in the current study. 1) In the control group, cardiomyocytes were treated with 0.05% DMSO for 3 h. 2) In the AlP group, cardiomyocytes were treated with 20 μg/ml of AlP (IC_50_ 3 h) for 3 h according to our previous study (Khezri et al., 2020). 3) In the AlP + Myr group, cardiomyocytes were cotreated with 20 μg/ml of AlP and 20 μM of Myr for 3 h. 4) In the AlP + Myr group, cardiomyocytes were cotreated with 20 μg/ml of AlP and 40 μM of Myr for 3 h. 5) In the AlP + Myr group, cardiomyocytes were cotreated with 20 μg/ml of AlP and 80 μM of Myr for 3 h. 6) In the Myr group, cardiomyocytes were treated with 80 μM of Myr for 3 h.

### Measurement of Cytotoxicity

Cytotoxicity was measured by MTT assay in a 96-well plate. After 3 h of exposure to AlP and Myr according to the above groupings, the isolated cardiomyocytes were treated with MTT at 0.5 mg/ml for 2 h. The purple formazan crystals were dissolved in 100 µl of DMSO, and the absorbance was measured at 570 nm. Five independent experiments were performed in triplicate (Khezri et al., 2020).

### Caspase-3 Activation

The activation of caspase-3 activity was measured using “Sigma’s caspase 3 assay kit” (CASP-3-C). Briefly, the hydrolysis of substrate peptide, Ac-DEVD-pNA, through caspase-3 in the base was assessed for caspase-3 activation. The released segment of *p*-nitroaniline has a high absorbance at 405 nm.

### ATP/ADP Ratio Assay

ADP/ATP ratio was assessed by ADP/ATP Ratio Assay kit (MAK135, Sigma, USA) in isolated cardiomyocytes using luminometer. ADP/ATP ratio was assessed according to the manufacturer’s instructions (Salimi et al., 2015).

### Detection of Reactive Oxygen Species in Cardiomyocytes

Intracellular reactive oxygen species (ROS) generation in cardiomyocytes was detected by staining with fluorescence dye DCFH-DA. Cardiomyocyte ROS level could be monitored by detecting of the fluorescence intensity of DCF, by using flow cytometry. Briefly, after 3 h of exposure to AlP and Myr according to the abovementioned experimental groups, the cardiomyocytes were washed twice with PBS and incubated with 5 µM of DCFH-DA dissolved in CCT medium for 15 min in a dark chamber. Then the fluorescence intensity of DCF was detected by flow cytometry (CyFlow Space-Partec, Sysmex Partec GmbH, Görlitz, Germany) and analyzed by FlowJo software (Eruslanov and Kusmartsev, 2010).

### Mitochondrial Membrane Potential Measurement

Mitochondrial membrane potential was measured with a unique cationic dye of rhodamine 123. Briefly, the cardiomyocytes were treated according to the experimental groups described above for 3 h with AlP and Myr. Then the cardiomyocytes were washed twice with PBS and incubated with 5 µM of rhodamine 123 dissolved in CCT medium for 15 min in a dark chamber. Then the fluorescence intensity of rhodamine 123 was detected by flow cytometry (CyFlow Space-Partec, Germany) and analyzed by FlowJo software (Khezri et al., 2020).

### Measurement of Lysosomal Membrane Integrity

Lysosomal membrane integrity was measured by staining with fluorescence dye AO. Briefly, the cardiomyocytes were treated according to the experimental groups described above for 3 h with AlP and Myr. Then the cardiomyocytes were washed twice with PBS and incubated with 5 µM of AO dissolved in CCT medium for 15 min in a dark chamber. Then the fluorescence intensity of AO was detected by flow cytometry (CyFlow Space-Partec, Germany) and analyzed by FlowJo software (Khezri et al., 2020).

### Determination of Reduced and Oxidized Glutathione Contents

The contents of GSH and oxidized glutathione (GSSG) were measured by the Hissin and Hilf method (Hissin and Hilf, 1976). Briefly, the cardiomyocytes were treated according to the experimental groups described above for 3 h with AlP and Myr. The cardiomyocytes were washed twice with PBS, resuspended in phosphate buffer (0.1 M with pH 7.4), and mechanically lysed using glass homogenizer. The cell lysate was centrifuged for 8,000 × *g* at 4°C for 10 min, and the supernatants were used for GSH and GSSG determination according to the Hissin and Hilf method 1976) using the enzymatic recycling method with DTNB and glutathione reductase (GR) in a microplate format using a plate reader. For detection of GSSG, 100 μl of supernatant was mixed to 3 ml of reaction solution (150 μM of NADPH, 500 mM of TRIS–HCl buffer, 1 mM of EDTA, glutathione reductase, 10 mM of DTNB, and 3 mM of MgCl_2_). Also, for detection of GSH, 100 μl of supernatant was mixed with 3 ml of reaction solution (500 mM of TRIS–HCl and 10 mM of DTNB with pH = 8.0). After 15 min of incubation at 25°C, the optical density was measured at 412 nm.

### Assay of Lipid Peroxidation in Cardiomyocytes

Lipid peroxidation was measured by production of thiobarbituric acid (TBA) reactive substances (TBARS). Briefly, the cardiomyocytes were treated according to the experimental groups described above for 3 h with AlP and Myr. The cardiomyocytes were washed twice with PBS and mechanically lysed in 1 ml of 0.1% (w/v) trichloroacetic acid (TCA) and centrifuged at 10,000 × *g* for 10 min. A volume of 200 µl of supernatant was mixed with 400 µl of 20% TCA and 0.5% TBA solution and then boiled at 95°C for 20 min. After cooling on ice and centrifugation at 1,000 × *g* for 10 min, the absorbance was measured at 532 nm (Beach and Giroux, 1992).

### Analysis of Antioxidant Enzymes in Cardiomyocytes

The cardiomyocytes were seeded in a 24-well plate at a density of 1 × 10^5^ cells/ml and treated according to the experimental groups described above for 3 h with AlP and Myr. Then, the cardiomyocytes were washed with PBS and centrifuged at 300 × *g* for 10 min. The collected cells were crushed by ultrasonic wave, and the cell lysates were resuspended. SOD, CAT, and GSH-Px activities were determined with a microplate reader according to the protocol of the detection kit. The activity of SOD was measured by the xanthine oxidase method. The activity of GSH-Px was detected by the colorimetric method. The activity of CAT activity was detected by the visible spectrophotometer method.

### Isolation of Mitochondria

Cardiac mitochondria were isolated from rat heart by differential centrifugation of homogenates with minor modification as described previously (Schulz et al., 2015). Briefly, after deep anesthesia was administered to the experimental animals, their hearts were explanted, washed with normal saline, and cut into mall slices by surgical scissors and cleared from extra tissues. The sliced tissues were homogenized in the isolation buffer (components are mentioned in *Solutions and Drugs* section) using a glass homogenizer, and then the cell lysates were centrifuged at 1,000 × *g* for 10 min. The pellet containing nuclei and undisrupted cells was removed, and the supernatant containing mitochondrial fraction were centrifuged again at 10,000 × *g* for 10 min at 4°C. All solutions and equipment were kept on ice bath during the isolation process. Mitochondria were stored in ice, and mitochondrial protein concentration was measured by the Bradford assay using serum albumin as standard (Mersa et al., 2020). The integrity and purity of isolated mitochondria were measured by succinate dehydrogenase (SDH) and lactate dehydrogenase (LDH) assays.

### Measurement of Mitochondrial Succinate Dehydrogenase Activity

The mitochondrial SDH activity was measured through MTT reduction at 570 nm. Briefly, isolated mitochondria (1,000 μg/ml) were incubated in a 96-well plate with total volume of 100 µl/well, in assay buffer, and treated according to the experimental groups described above for 1 h with AlP and Myr. After incubation, 0.4% MTT was added and incubated at 37°C for 30 min. Finally, the formazan crystals were dissolved in 100 µl of DMSO, and the optical density was measured at 570 nm (Mersa et al., 2020).

### Measurement of Mitochondrial Swelling

Mitochondrial swelling was measured with a plate reader (BioTek, Winooski, VT, USA) that measured absorbance at 540 nm in a swelling buffer. Briefly, isolated mitochondria (1,000 μg/ml) were incubated in a 96-well plate with total volume of 100 µl/well, in swelling buffer (140 mmol/L of KCl, 2 mmol/L of MgCl_2_, 10 mmol/L of NaCl, 0.5 mmol/L of KH_2_PO_4_, 20 mmol/L of HEPES, and 0.5 mmol/L of EGTA and supplemented with 1 mg/ml of rotenone, and pH was adjusted to 7.4) and treated according to the experimental groups described above for 1 h with AlP and Myr. The absorbance of the samples was monitored for 1 h in 15-min intervals at 540 nm. Reduction of absorbance is related to increase in mitochondrial swelling (Zhao et al., 2010).

### Data Analysis

Differences between groups were assessed using the one-way ANOVAs and two-way ANOVAs followed by post-hoc Tukey’s and Bonferroni’s tests, respectively (GraphPad Prism 5; GraphPad Software, San Diego, CA, USA). Values are shown as means ± SD. *p* ≤ 0.05 was considered significant.

## Results

### Ameliorative Efficacy of Myricetin in Aluminum Phosphide-Induced Cytotoxicity in Cardiomyocytes

**FIGURE 1:**
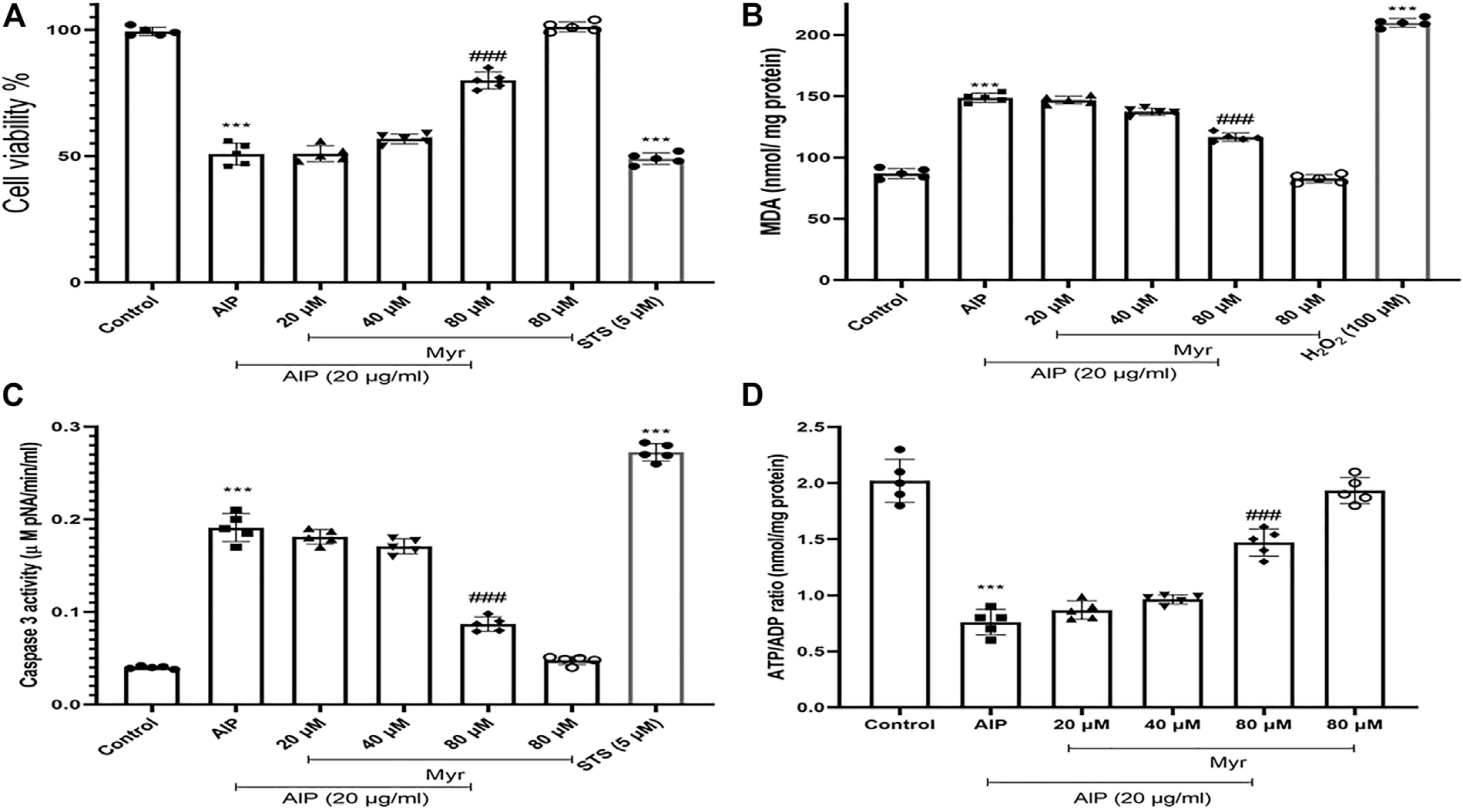
Ameliorative effect of Myr on AlP-induced cytotoxicity in isolated cardiomyocytes. **(A)** Isolated cardiomyocytes were treated or cotreated with the indicated concentrations (20 μg/ml) of AlP and Myr (20, 40, and 80 µM) for 3 h. Cell viability was examined after incubation using MTT assay. Myr inhibits AlP-induced cytotoxicity in isolated cardiomyocytes. **(B)** Isolated cardiomyocytes were treated or cotreated with Myr (20, 40, and 80 μM) and AlP (20 μg/ml) for 3 h. MDA production was measured by production of thiobarbituric acid (TBA) reactive substances (TBARS). **(C)** Isolated cardiomyocytes (10^6^ cells/ml) were incubated in CCT medium in conventional condition (37°C and 5% CO_2_-air) for 3 h. Caspase-3 activity was determined by Sigma-Aldrich kit. Columns represent caspase-3 activity (μM pNA/min/ml) in isolated cardiomyocytes. **(D)** ATP/ADP ratios were determined by Luciferin/Luciferase assay as described in the *Materials and Methods*. Values represent mean ± SD (*n* = 3) of three independent experiments. ***p < 0.001 versus control; ###*p* < 0.001 versus AlP-treated cardiomyocytes, one-way ANOVA, Tukey’s test. Myr, myricetin; AlP, aluminum phosphide; MDA, malondialdehyde; STS, staurosporine; CCT, creatine–carnitine–taurine.

To determine the cytotoxicity of AlP and the ameliorative effect of Myr on isolated cardiomyocytes, the cell viability was evaluated after 3-h exposure by MTT assay. Cell viability of isolated cardiomyocytes markedly decreased following incubation with 20 μg/ml of AlP. To evaluate the ameliorative effect of Myr in AlP-induced cytotoxicity, isolated cardiomyocytes were cotreated for 3 h with the indicated concentrations of Myr. The results of the MTT assay following 80 µM of Myr cotreatment indicated a significantly increased cell viability as compared with cells treated with AlP alone. Cotreatment with 80 μM of Myr increased the cell viability to 81% ± 4.2% (Figure 1A). As positive control for cell death, 5 μM of staurosporine (STS) was used.

### Myricetin Inhibits Aluminum Phosphide-Induced Malondialdehyde Production

Malondialdehyde (MDA) as an end product of lipid oxidation is considered to be a reliable indicator of ROS formation and oxidative stress. The MDA levels were measured as previously described in the *Materials and Methods* section to investigate the effect of Myr on AlP-induced lipid peroxidation. A significant elevation of the MDA contents was observed in isolated cardiomyocytes with 20 μg/ml of AlP compared with the control group, whereas cotreatment with 80 µM of Myr exhibited a significant decrease in lipid peroxidation (Figure 1B). The results showed that cotreatment of isolated cardiomyocytes with Myr inhibited AlP-induced MDA production, alleviated lipid peroxidation of the cell membrane, and reduced cell damage. Hydrogen peroxide (H_2_O_2_) was used as a positive control (100 µM).

### Myricetin Inhibits Aluminum Phosphide-Induced Caspase-3 Activation

As shown in Figure 1C, activation of caspase-3 was observed following treatment of isolated cardiomyocytes with AlP, while Myr (80 µM) significantly reduced AlP-induced caspase-3 activation in the isolated cardiomyocytes. As positive control for caspase-3 activation, 5 μM of STS was used.

### Myricetin Inhibits Aluminum Phosphide-Induced ATP Depletion

ATP/ADP ratio was assessed by ADP/ATP Ratio Assay kit (MAK135, Sigma, USA) in isolated cardiomyocytes using luminometer. As shown in Figure 1D, ATP/ADP ratio significantly (*p* < 0.001) decreased by AlP, while Myr significantly inhibited AlP-ATP depletion in the isolated cardiomyocytes (Figure 1D).

### Myricetin Inhibits Aluminum Phosphide-Induced Reactive Oxygen Species Formation

**FIGURE 2:**
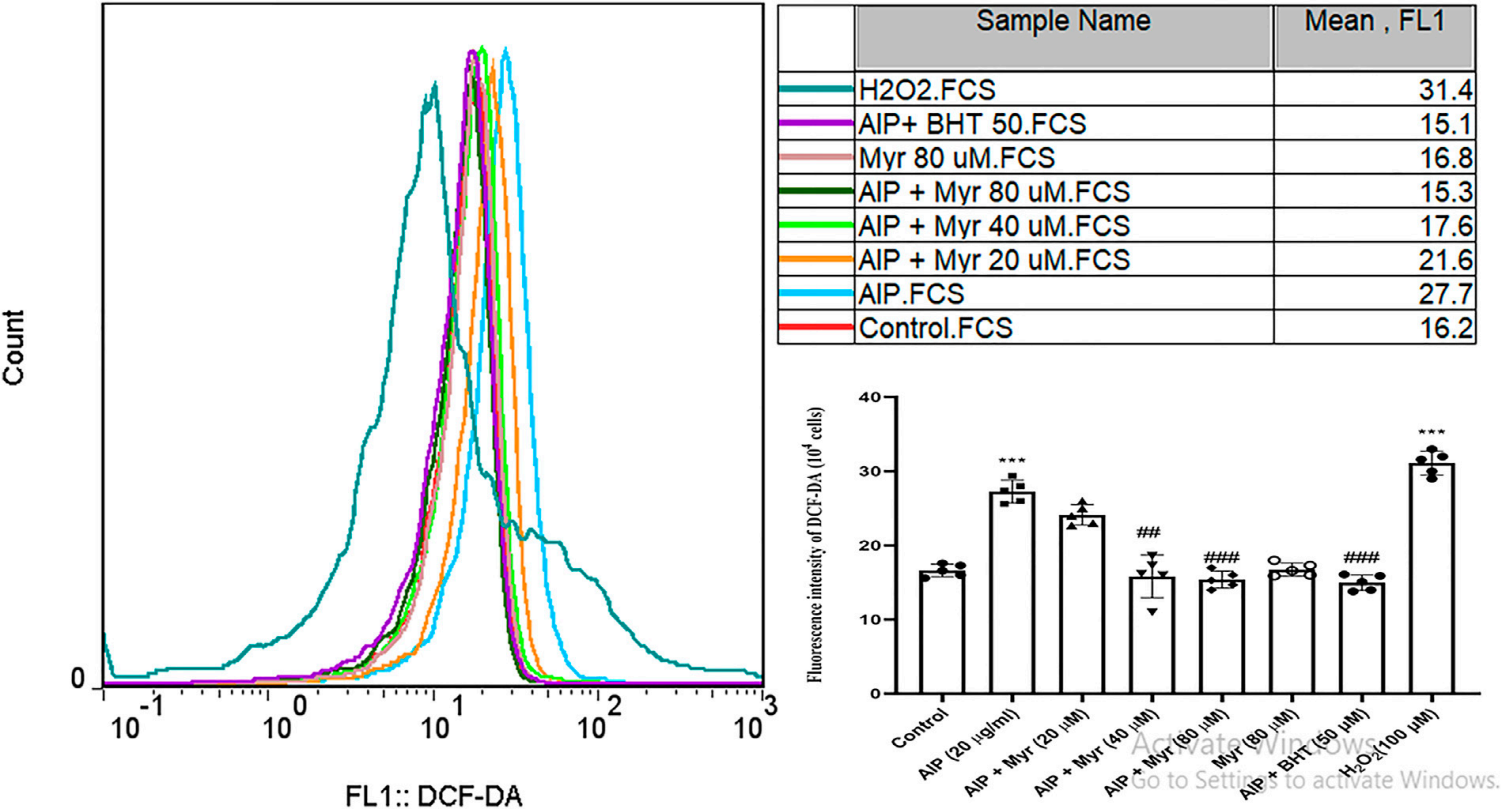
Myr inhibits AlP-induced ROS production in isolated cardiomyocytes. As shown, the fluorescence intensity of DCF is increased after exposure to AlP. The peak is moved to the right as compared with control, while cotreatment of Myr (40 and 80 µM) with AlP showed that the fluorescence intensity of DCF is decreased, and the peaks are moved to the left. Values represent mean ± SD (*n* = 3) of three independent experiments, ***p < 0.001 compared with control; ##*p* < 0.01; ###*p* < 0.001 compared with AlP-treated cardiomyocytes, one-way ANOVA, Tukey’s test. Myr, myricetin; AlP, aluminum phosphide; ROS, reactive oxygen species; DCF-DA, 2ʹ,7ʹ-dichlorofluorescin diacetate; BHT, butylated hydroxytoluene; H_2_O_2_, hydrogen peroxide.

Intracellular ROS by AlP-induced was monitored by DCFH-DA in isolated cardiomyocytes using flow cytometry. As shown in Figure 2, incubation with AlP for 3 h led to an increase in DCF fluorescence intensity and a shift of DCF peak rightward, which is proportionate to the amount of ROS generated. The result showed that exposure to 20 μg/ml of AlP increased intracellular ROS generation in isolated cardiomyocytes and shifted the peak rightward as compared with the untreated cardiomyocytes. However, cotreatment with Myr (40 and 80 µM) effectively reduced AlP-induced ROS production, as evidenced by the lower DCF fluorescence intensity in Myr-cotreated cardiomyocytes, and shifted the peak leftward as compared with AlP group alone. BHT (50 µM), a known antioxidant, was added to verify that the antioxidant effect of Myr inhibits ROS formation. H_2_O_2_ was used as a positive control (100 µM).

### Myricetin Inhibits Aluminum Phosphide-Induced Mitochondrial Membrane Potential Collapse

**FIGURE 3:**
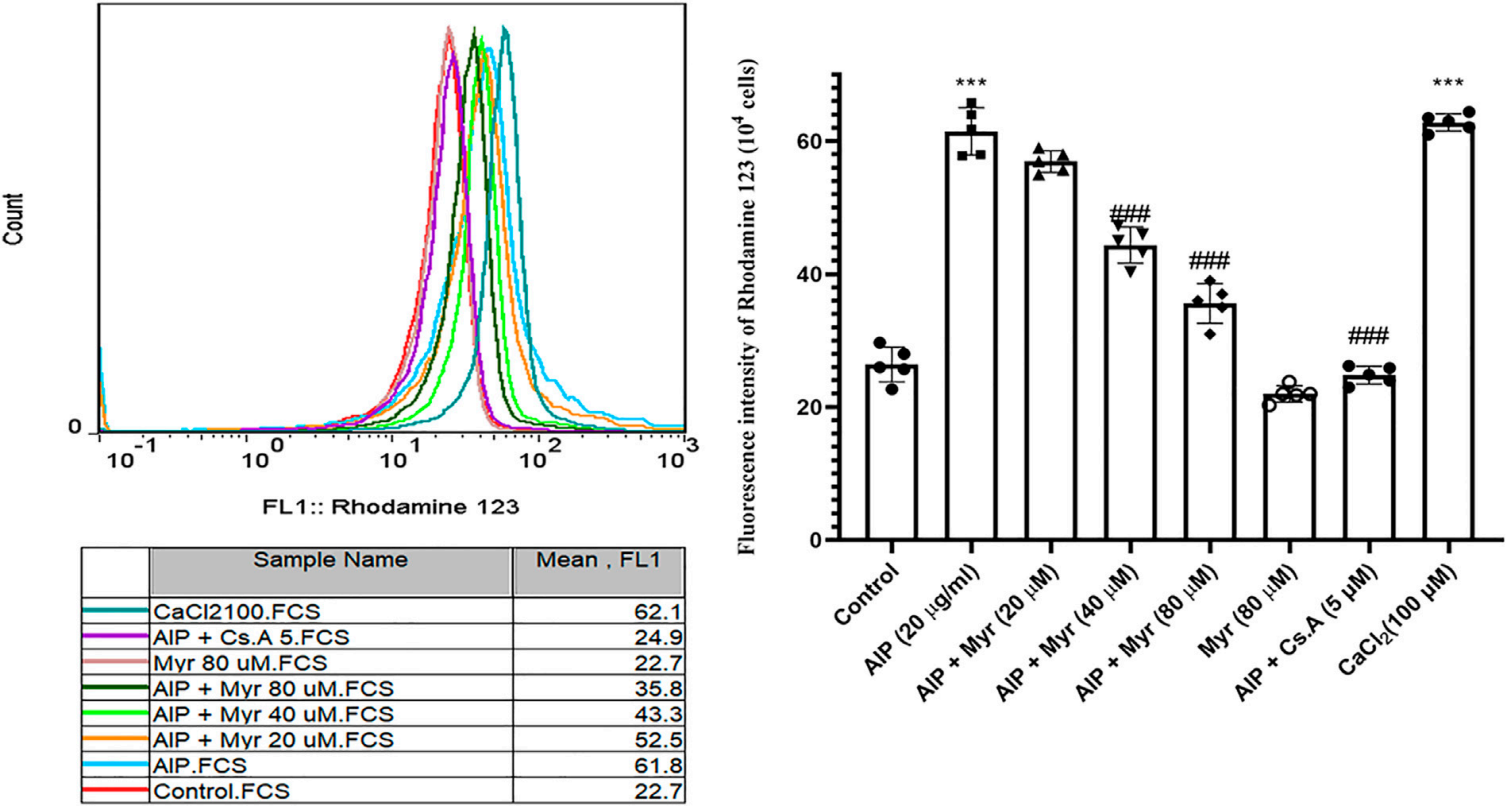
Myr inhibits AlP-induced mitochondrial membrane potential (ΔΨm) collapse in isolated cardiomyocytes. Isolated cardiomyocytes were treated or cotreated with the indicated concentrations (20 μg/ml) of AlP and Myr (20, 40, and 80 µM) for 3 h. Mitochondrial membrane potential (ΔΨm) collapse was examined after incubation using rhodamine 123 staining. Representative fluorescence intensity of rhodamine 123 staining. Data presented are the mean ± SD (*n* = 3 per group). ***p < 0.001 compared with control; ##*p* < 0.01; ###*p* < 0.001 compared with AlP-treated cardiomyocytes, one-way ANOVA, Tukey’s test. Myr, myricetin; AlP, aluminum phosphide; Cs.A, cyclosporine; CaCl₂, calcium chloride.

Mitochondrial membrane potential collapse by AlP-induced was monitored by rhodamine 123 in isolated cardiomyocytes using flow cytometry. As shown in Figure 3, incubation with AlP for 3 h led to an increase in rhodamine 123 fluorescence intensity, which is proportionate to collapse of mitochondrial membrane potential. The result demonstrated that exposure to 20 μg/ml of AlP increased the mean of rhodamine 123 fluorescence intensity in isolated cardiomyocytes compared with the untreated cardiomyocytes. However, cotreatment with Myr (40 and 80 µM) effectively reduced AlP-induced mitochondrial membrane potential collapse, as evidenced by the lower rhodamine 123 fluorescence intensity in Myr-cotreated cardiomyocytes compared with AlP group alone. Cyclosporine A (5 µM), a PTP inhibitor, was added to verify PTP dependence of mitochondrial swelling. CaCl_2_ (100 µM), a known inducer of mitochondrial permeability transition (MPT), was used as a positive control.

### Myricetin Inhibits Aluminum Phosphide-Induced Lysosomal Damages

**FIGURE 4:**
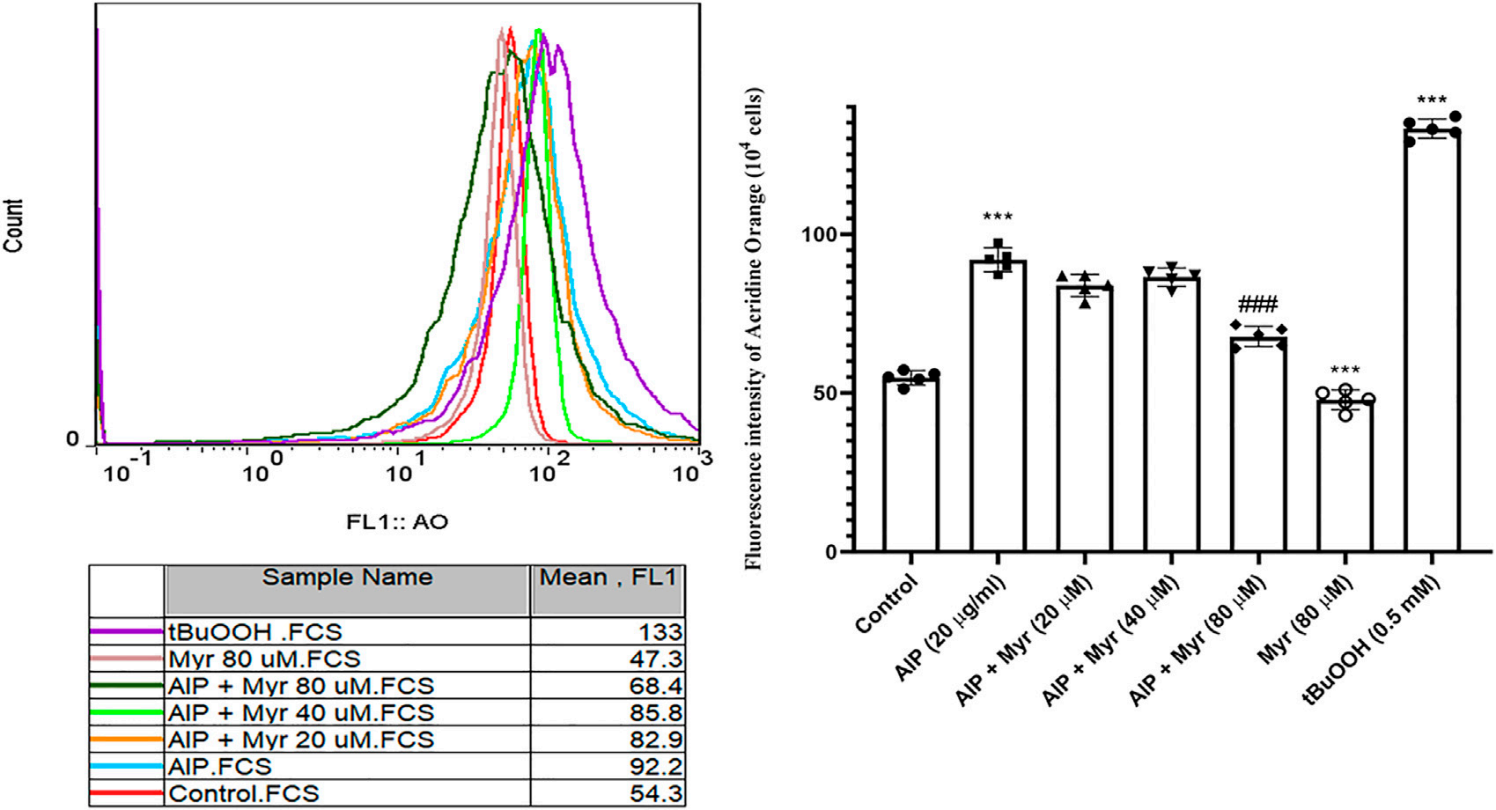
Myr inhibits AlP-induced lysosomal dysfunction in isolated cardiomyocytes. Isolated cardiomyocytes were treated or cotreated with the indicated concentrations (20 μg/ml) of AlP and Myr (20, 40, and 80 µM) for 3 h. Lysosomal membrane stability was examined after incubation using acridine orange fluorescence dye. Data are mean ± SD (*n* = 3) of three independent experiments. ***p < 0.001 significantly different from control, ##*p* < 0.01 significantly different from AlP-treated cardiomyocytes, one-way ANOVA, Tukey’s test. Myr, myricetin; AlP, aluminum phosphide; AO, acridine orange; *t*-BuOOH, *tert*-butyl hydroperoxide.

AlP-induced lysosomal membrane destabilization was monitored by AO in isolated cardiomyocytes using flow cytometry. As showed in Figure 4, incubation with AlP for 3 h led to an increase in AO fluorescence intensity, which is proportionate to lysosomal damages. The result indicated that exposure to 20 μg/ml of AlP increased the mean of AO fluorescence intensity in isolated cardiomyocytes compared with the untreated cardiomyocytes. However, cotreatment with Myr (40 and 80 µM) effectively reduced AlP-induced lysosomal membrane destabilization, as evidenced by the lower AO fluorescence intensity in Myr-cotreated cardiomyocytes compared with AlP group alone. *tert*-Butyl hydroperoxide (*t*-BuOOH) at concentration of 0.5 mM, a classical lysosomal membrane permeabilization inducer that causes lysosomal damage via oxidative stress, was used as positive control.

### Ameliorative Effects of Myricetin on Aluminum Phosphide-Induced Glutathione Depletion

**FIGURE 5:**
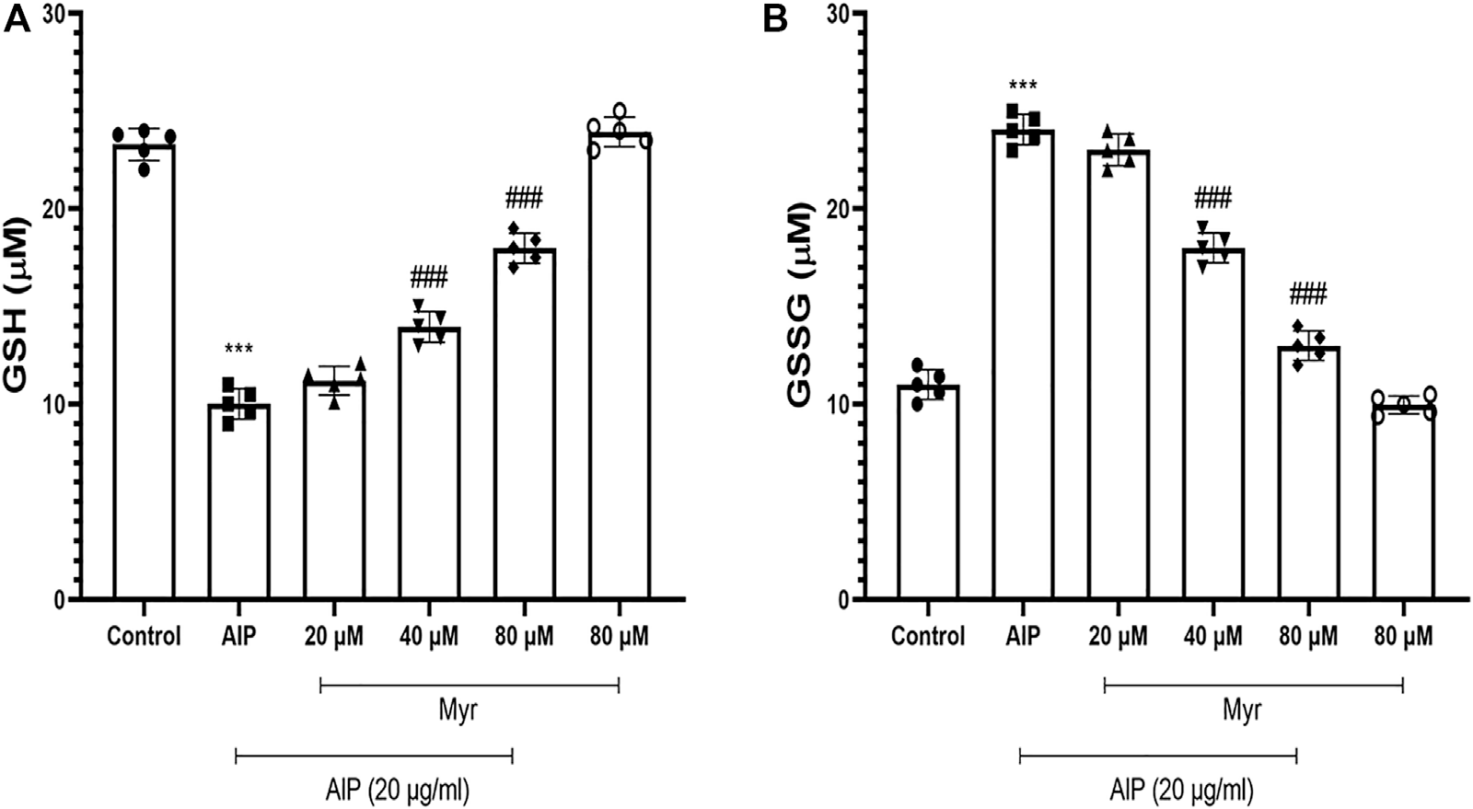
Myr inhibits AlP-induced GSH depletion in isolated cardiomyocytes. Isolated cardiomyocytes were treated or cotreated with the indicated concentrations (20 μg/ml) of AlP and Myr (20, 40, and 80 µM) for 3 h. GSH and GSSG levels were measured as previously described in the *Materials and Methods* section. **(A)** GSH content significantly decreased AlP-treated isolated cardiomyocytes, while cotreatment of Myr (40 and 80 µM) with AlP obviously increased the GSH content in isolated cardiomyocytes. **(B)** AlP significantly increased the GSSG content in isolated cardiomyocytes after 3 h of exposure, while Myr (40 and 80 µM) significantly decreased the GSSG content in the presence of AlP. Data are mean ± SD (*n* = 3) of three independent experiments. ***p < 0.001 significantly different from control, ###*p* < 0.001 significantly different from AlP-treated cardiomyocytes, one-way ANOVA, Tukey’s test. Myr, myricetin; AlP, aluminum phosphide; GSH, reduced glutathione; GSSG; oxidized glutathione.

Since AlP is a glutathione-depleting agent, we investigated the effects of AlP and Myr on GSH level by measuring the GSH and GSSG. A significant reduction in GSH was observed after AlP treatment in isolated cardiomyocytes. Myr cotreatment (40 and 80 µM) caused a significant recovery in the GSH level (Figure 5A). The GSSG levels was significantly increased in AlP-treated cardiomyocytes. Myr cotreatment (40 and 80 µM), however, caused a significant reduction in the GSSG level (Figure 5B). The results showed that Myr at 40 and 80 μM significantly prevented (*p* < 0.001) the depletion of GSH level caused AlP by in isolated cardiomyocytes.

### Effect of Aluminum Phosphide and Myricetin on Antioxidant Enzyme in Cardiomyocytes

To determine whether AlP and Myr have effects on the levels of antioxidant enzymes, GSH-Px, CAT, and SOD were measured in the cell lysates. The activities of GSH-Px, CAT, and SOD were remarkably decreased in the AlP group compared with the control group (*p* < 0.01). Myr cotreatment (80 µM) with AlP (20 μg/ml) could increase the activities of SOD, CAT, and GSH-Px compared with that in the AlP group (*p* < 0.01) (Table 1).

**TABLE 1:** Effects of AlP at concentration 20 μg/ml and Myr at concentrations of 20, 40, and 80 µM + AlP on superoxide dismutase (SOD), catalase (CAT), and glutathione peroxidase (GSH-Px) levels of rat heart isolated cardiomyocytes at 3 h.

Groups	SOD (U/L)	CAT (U/mg protein)	GSH-Px (U/mg protein)
	3 h	3 h	3 h
Control	245 ± 7.5	16.1 ± 0.6	128 ± 5.6
AlP (20 μg/ml)	158 ± 9.1^a^	7.3 ± 0.3^a^	36 ± 4.4^a^
AlP + Myr (20 µM)	159 ± 6.4	7.1 ± 0.2	37 ± 2.3
AlP + Myr (40 µM)	163 ± 7.9	8.3 ± 0.4	36 ± 3.4
AlP + Myr (80 µM)	179 ± 8.3^b^	13.5 ± 0.7^b^	89 ± 2.2^b^
Myr (80 µM)	249 ± 5.3	17.1 ± 0.2	127 ± 4.2

Note. AlP, aluminum phosphide; Myr, myricetin.

a Shows significant difference (*p* < 0.05) with control.

b Shows significant difference (*p* < 0.05) with AlP-treated group with 20 μg/ml.

### Ameliorative Efficacy of Myricetin in Aluminum Phosphide-Induced Mitochondrial Dysfunction

**FIGURE 6:**
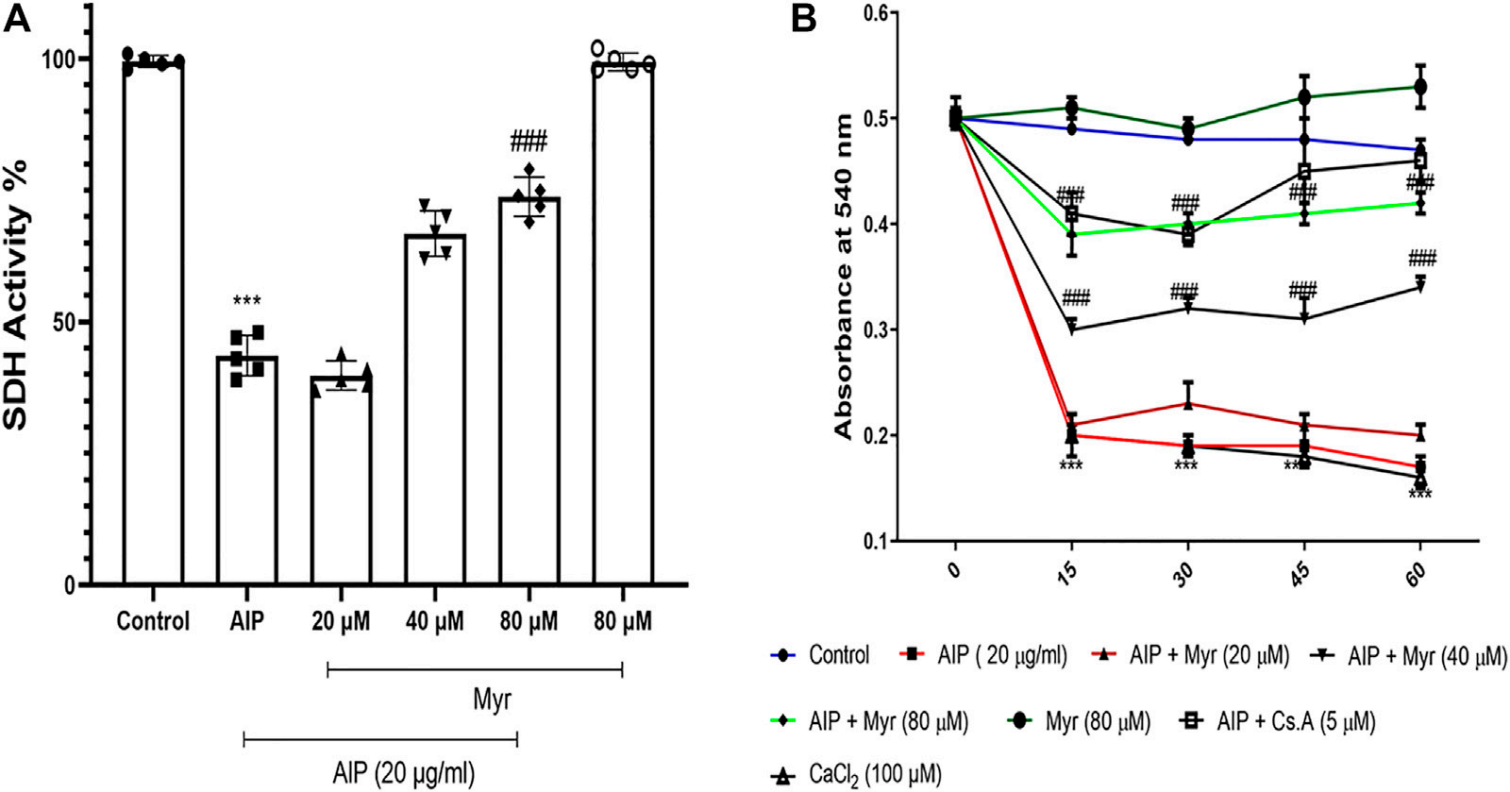
Myr inhibits AlP-induced mitochondrial dysfunction **(A)** and swelling **(B)** in isolated mitochondria. **(A)** Isolated mitochondria were treated or cotreated with the indicated concentrations (20 μg/ml) of AlP and Myr (20, 40 and 80 µM) for 1 h. Succinate dehydrogenase activity was evaluated by MTT assay. Data showed AlP (20 μg/ml) to significantly decrease SDH activity as compared with control, while Myr (40 and 80 µM) significantly increased SDH activity as compared with AlP-treated cardiomyocytes. **(B)** Isolated mitochondria were treated or cotreated with the indicated concentrations (20 μg/ml) of AlP and Myr (20, 40, and 80 µM) for 1 h. Mitochondrial swelling was evaluated by monitoring absorbance at 540 nm. Data showed that AlP (20 μg/ml) significantly induced mitochondrial swelling as compared with control, while Myr (40 and 80 µM) significantly inhibits mitochondrial swelling as compared with AlP-treated cardiomyocytes. Data are mean ± SD (*n* = 3) of three independent experiments. ***p < 0.001 significantly different from control, ##*p* < 0.01, ###*p* < 0.001 significantly different from AlP-treated cardiomyocytes, one-way ANOVA and two-way ANOVA followed by post-hoc Tukey and Bonferroni test. Myr, myricetin; AlP, aluminum phosphide; SDH, succinate dehydrogenase activity.

To determine the mitochondrial dysfunction of AlP and the ameliorative effect of Myr on isolated mitochondria, the SDH activity was evaluated after 1-h exposure by MTT assay. The SDH activity in isolated mitochondria markedly decreased following incubation with 20 μg/ml of AlP. To evaluate the ameliorative effect of Myr in AlP-induced mitochondrial dysfunction, isolated mitochondria were cotreated for 1 h with the indicated concentrations of Myr (20, 40, and 80 μM). The results of the SDH activity following 40 and 80 µM of Myr cotreatment indicated a significant increase of the mitochondrial activity compared with mitochondria treated with AlP alone. Cotreatment with 80 and 40 μM of Myr increased the mitochondrial activity to 67% ± 5.1% and 74% ± 4.9%, respectively, as compared with mitochondria treated with AlP alone (Figure 6A).

### Myricetin Inhibits Aluminum Phosphide-Induced Mitochondrial Swelling

We monitored the ameliorative effects of Myr against AlP-induced mitochondrial swelling as an indicator of MPT pore opening. To determine the mitochondrial swelling of AlP and the ameliorative effect of Myr on isolated mitochondria, the absorbance at 540 nm was evaluated during 1-h exposure. The mitochondrial swelling markedly increased following incubation with 20 μg/ml of AlP. To evaluate the ameliorative effect of Myr in AlP-induced mitochondrial swelling, isolated mitochondria were cotreated for 1 h with the indicated concentrations of Myr (20, 40, and 80 μM). The results of the mitochondrial swelling following 40 and 80 µM of Myr cotreatment indicated a significant decrease of the mitochondrial swelling compared with mitochondria treated with AlP alone (Figure 6B). Cyclosporine A (5 µM), a MPT pore (PTP) inhibitor, was added to verify PTP dependence of mitochondrial swelling. CaCl_2_ (100 µM), a known inducer of MPT, was used as a positive control.

## Discussion

There are many investigations that showed the underlying mechanisms of AlP-induced toxicity. Mitochondrial dysfunction and oxidative stress are the major mechanisms in performed experimental studies (Valmas et al., 2008; Kariman et al., 2012; Anand et al., 2013; Sciuto et al., 2016). Oxidative stress and mitochondrial damages induced by AlP have been demonstrated in nematodes, insects, mammalian cell lines, and animals (Valmas et al., 2008; Sciuto et al., 2016). Although the underlying mechanisms of AlP is not well understood, it has been found that phosphine inhibits aerobic respiration in a number of tissues and species (Anand et al., 2013). Previous studies on submitochondrial particles and isolated mitochondria have disclosed that mitochondrial complex IV (cytochrome *c* oxidase) of the electron transport chain is inhibited by AlP (Anand et al., 2013; Sciuto et al., 2016). Our results on isolated cardiomyocytes and mitochondria showed that AlP induces mitochondrial toxicity and oxidative stress. Our findings are in accordance with previous reports that showed AlP that causes oxidative damages and mitochondrial dysfunction (Jahedsani et al., 2020; Khezri et al., 2020).

Antioxidants and mitochondrial protective agents are the simple and most significant defense system of the human body to counteract oxidative stress and mitochondrial dysfunction (Kurutas, 2015). The nonenzymatic agents including glutathione and enzymatic antioxidants such thiol-containing enzymes, SOD, and CAT are of great importance in the human body (Kurutas, 2015). Our results in the current study are in accordance with previous reports that have shown that AlP reduces the antioxidant molecules such as glutathione (Hsu et al., 2000). The action of AlP in the different tissues and species reported in previous studies and isolated cardiomyocytes in our study correlates well with depletion of glutathione, caspase-3 activation, ROS formation, and lipid peroxidation (Kariman et al., 2012). Decreased glutathione in isolated cardiomyocytes strongly suggested the involvement of ROS formation and lipid peroxidation in AlP cytotoxicity. Depletion of glutathione in the cardiomyocytes predisposes cells to oxidant damage, lipid peroxidation, and cytotoxicity (Mohamed et al., 2000). It has been proved that antioxidants such as melatonin can stop most of the oxidative damage induced by AlP in rat cardiac tissues and proprietorially preserve the levels of glutathione and mitochondrial function (Asghari et al., 2017). Other antioxidants such as *N*-acetylcysteine (NAC), which have antioxidant properties and reload cellular glutathione, have been proposed to reduce the cardiac toxicity induced by AlP (Tehrani et al., 2013). Sine myocardial suppression is a distinguishing feature of AlP poisoning via mitochondrial damages and oxidative stress, the simple and most significant strategy to reduce AlP-induced cardiotoxicity is to use antioxidant and mitochondrial protective agents (Akkaoui et al., 2007). These antioxidant compounds can reduce the toxicity of AlP, either directly or by reloading antioxidant defenses such as glutathione.

In various cell-based assays and animal models, the antioxidant effect of Myr was demonstrated. It has been reported that Myr has a protective effect through inactivation of H_2_O_2_-induced radicals as well as regulation of programmed cell death or apoptosis (Mansuri et al., 2014). Likewise, Myr reduced oxidative stress induced by hydrogen peroxide yeast cells and led to a reduction in protein carbonylation and intracellular oxidation (Mansuri et al., 2014). The beneficial effects of Myr on vascular endothelial dysfunction have been reported in human umbilical vein endothelial cells (Yi et al., 2011). Also, in several animal models, the antioxidant effect of Myr was also observed. It has been reported that Myr decreases the generation of myeloperoxidase, MDA, and nitric oxide while increasing the activity of glutathione peroxide and SOD in animal models (Zhao et al., 2013). Moreover, the antioxidant effects of Myr on cardiovascular function have been reported in animal models. Tiwari et al. have reported that Myr significantly inhibits the effects of histopathological alterations of ISO on heart rate, the levels of different cardiac marker enzymes, including aspartate aminotransferase (AST), creatine kinase (CK), LDH, CAT, and SOD as well alterations in electrocardiographic patterns and vascular reactivity in Wistar rats (Tiwari et al., 2009). Our results on isolated cardiomyocytes showed that Myr can inhibit oxidative stress, ROS formation, and depletion of glutathione and ATP and can increase the activities of SOD, CAT, and GSH-Px, which have the main role in AlP-induced cardiotoxicity. These results are in accordance with previous reports in cellar and animal studies (Tiwari et al., 2009; Yi et al., 2011; Taheri et al., 2020).

In various cell-based assays and animal models, the mitochondrial protective effects of Myr were demonstrated. The protective effects of Myr have been reported in preventing methylmercury-induced mitochondrial toxicity by blocking ROS formation and lipid peroxidation (Franco et al., 2010). Also in animal models, the mitochondrial protective effects of Myr on hypoxia-induced mitochondrial impairments were reported, and Myr attenuated acute hypoxia-induced mitochondrial impairment (Zou et al., 2015). At the mitochondrial level, AlP can rapidly inhibit oxidative respiration by up to 70% and perturb mitochondrial conformation, severely decreasing mitochondrial membrane potential (Valmas et al., 2008). It has been reported that AlP mainly inhibits complex IV and decreases complex I and complex II activity, resulting in decreased ATP production and increased ROS generation (Dua and Gill, 2004). These studies are in accordance with our results on isolated mitochondria in the current study (Dua and Gill, 2004). On the other hand, it has been proved that flavonoids such as Myr can suppress mitochondrial ROS production by directly chelating the trace elements and inhibiting enzymes (mitochondria complexes) involved in ROS formation (Kicinska and Jarmuszkiewicz, 2020). The Myr analogs are acacetin, chrysin, apigenin, luteolin, kaempferol, naringenin, and quercetin, which have shown similar effects (Taheri et al., 2020). Recently, we showed that apigenin and chrysin have a similar effect on isolated cardiomyocytes and mitochondria against AlP (Jahedsani et al., 2020; Khezri et al., 2020). It has been reported that quercetin, a very similar analog to Myr, can act as an inhibitor of the MPT pore, and the same effect of Myr was proved in this study (De Marchi et al., 2009). On the other hand, it has been reported that these inhibitors of the MPT pore can play an effective role in reducing myocardial damage (Hausenloy et al., 2002).

In the current study, we proved that Myr can inhibit mitochondrial dysfunction induced by AlP in isolated mitochondria and cardiomyocytes, resulting in decreased ROS generation, MDA level, and lysosomal damages and increased cell viability. For the futures studies, it is suggested that more researches be done on the effect of Myr on AlP-induced cardiotoxicity, and the findings of this study should be confirmed by animal and human studies.

In conclusion, the results of current study demonstrated that AlP can directly cause toxicity in cardiac mitochondria and cardiomyocytes, which are associated with cytotoxicity, mitochondrial toxicity, reduction of antioxidant molecules, ROS formation, oxidative stress, and lysosomal dysfunction, which significantly attenuated by Myr as an antioxidant and mitochondrial protective agent in isolated cardiomyocytes and mitochondria. In the light of these findings, we concluded that Myr through antioxidant potential and inhibition of MPT pore exerted an ameliorative role in AlP-induced toxicity in isolated cardiomyocytes and mitochondria, and it would be valuable to examine *in vivo* effects.

## Data Availability

The original contributions presented in the study are included in the article/Supplementary Material, Further inquiries can be directed to the corresponding author.
