# Supplementary figures and images for: Antioxidant Potential and Inhibition of Mitochondrial Permeability Transition Pore by Myricetin Reduces Aluminium Phosphide-Induced Cytotoxicity and Mitochondrial Impairments
Source: Front Pharmacol. 2021 Nov 9;12:719081. doi: 10.3389/fphar.2021.719081 (PMC8630626; doi:10.3389/fphar.2021.719081)

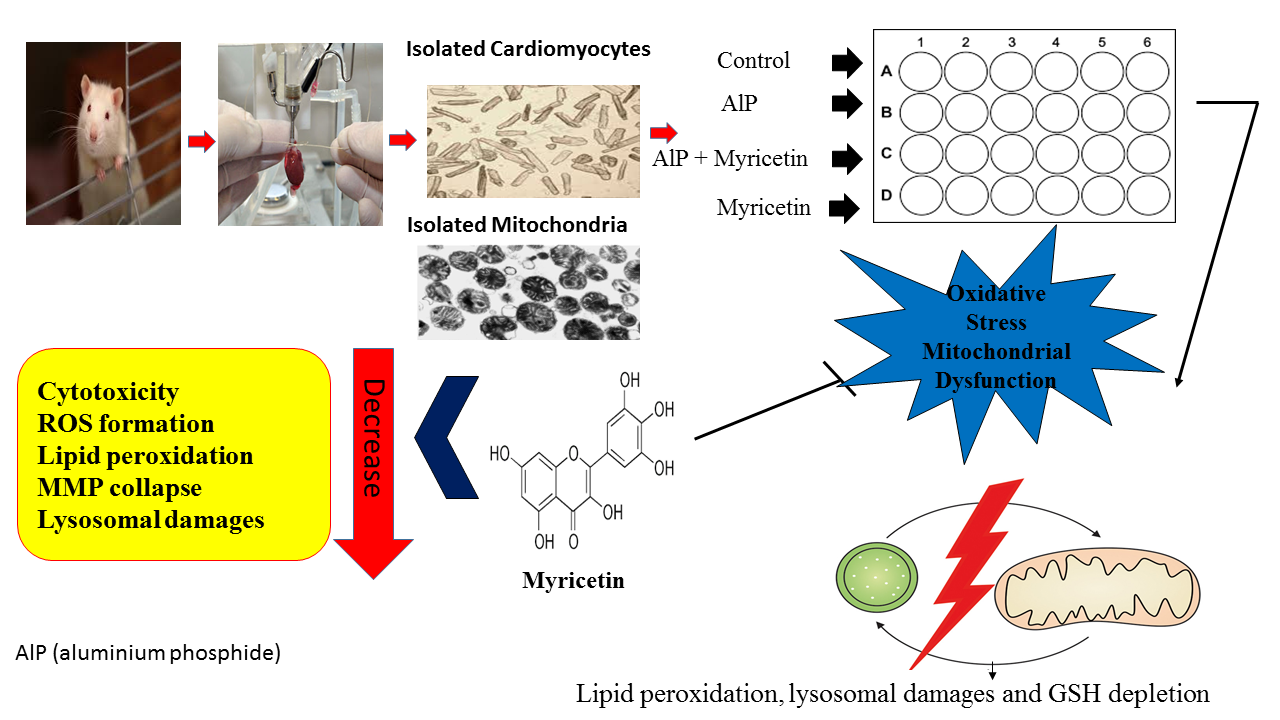

Supplement: Supplementary file 1 [file Image1.TIF]
